# Positron Emission Tomography (PET) Radiopharmaceuticals in Multiple Myeloma

**DOI:** 10.3390/molecules25010134

**Published:** 2019-12-29

**Authors:** Christos Sachpekidis, Hartmut Goldschmidt, Antonia Dimitrakopoulou-Strauss

**Affiliations:** 1 Clinical Cooperation Unit Nuclear Medicine, German Cancer Research Center, 69120 Heidelberg, Germany; a.dimitrakopoulou-strauss@dkfz.de; 2 Department of Internal Medicine V, University Hospital Heidelberg and National Center for Tumor Diseases (NCT), 69120 Heidelberg, Germany; Hartmut.Goldschmidt@med.uni-heidelberg.de

**Keywords:** multiple myeloma, positron emission tomography/computed tomography, radiopharmaceuticals, ^18^F-fluorodeoxyglucose

## Abstract

Multiple myeloma (MM) is a plasma cell disorder, characterized by clonal proliferation of malignant plasma cells in the bone marrow. Bone disease is the most frequent feature and an end-organ defining indicator of MM. In this context, imaging plays a pivotal role in the management of the malignancy. For several decades whole-body X-ray survey (WBXR) has been applied for the diagnosis and staging of bone disease in MM. However, the serious drawbacks of WBXR have led to its gradual replacement from novel imaging modalities, such as computed tomography (CT), magnetic resonance imaging (MRI) and positron emission tomography/computed tomography (PET/CT). PET/CT, with the tracer ^18^F-fluorodeoxyglucose (^18^F-FDG), is now considered a powerful diagnostic tool for the detection of medullary and extramedullary disease at the time of diagnosis, a reliable predictor of survival as well as the most robust modality for treatment response evaluation in MM. On the other hand, ^18^F-FDG carries its own limitations as a radiopharmaceutical, including a rather poor sensitivity for the detection of diffuse bone marrow infiltration, a relatively low specificity, and the lack of widely applied, established criteria for image interpretation. This has led to the development of several alternative PET tracers, some of which with promising results regarding MM detection. The aim of this review article is to outline the major applications of PET/CT with different radiopharmaceuticals in the clinical practice of MM.

## 1. Introduction

Multiple myeloma (MM) is a neoplastic plasma cell disorder, characterized by the uncontrolled, clonal proliferation of plasma cells in the bone marrow. It is the second most common hematologic malignancy after non-Hodgkin’s lymphoma accounting for approximately 1% of neoplastic diseases, and the most common primary tumor of the skeleton [1]. MM is almost always preceded from a premalignant precursor condition (monoclonal gammopathy of undetermined significance, MGUS), which then develops into asymptomatic or smoldering myeloma (SMM) and, finally, into symptomatic disease [2]. Bone involvement in the form of focal osteolytic lesions—the hallmark radiographic sign of MM—represents a marker of disease-related end-organ damage, necessitating immediate initiation of treatment [3]. Bone disease is a major cause of morbidity and mortality for patients suffering from MM. Since practically all patients develop bone involvement during the course of the disease [4], its reliable identification represents a pivotal diagnostic challenge. Historically, skeletal damage has been assessed by conventional, whole-body X-ray survey (WBXR), which was the standard imaging approach for MM. Nevertheless, this modality carries several limitations, including a low sensitivity—requiring a more than 30% bone demineralization before an osteolytic lesion becomes evident—its failure to detect extramedullary disease (EMD), which is a significant adverse prognostic factor of MM, and its poor performance in treatment response assessment [5]. The drawbacks of planar radiography have been overcome in recent years with the development and introduction in clinical practice of myeloma of novel imaging modalities, namely whole-body computed tomography (CT), magnetic resonance imaging (MRI) and positron emission tomography/computed tomography (PET/CT). These techniques offer a higher sensitivity than WBXR, leading to its gradual substitution by them.

It is undisputable that the role of PET/CT with the radiotracer ^18^F-fluorodeoxyglucose (^18^F-FDG) in MM has been upgraded with an increasing amount of literature highlighting its value in diagnosis, prognosis and treatment response evaluation of the disease. According to the latest update of the International Myeloma Working group (IMWG), the detection of one or more osteolytic lesions on CT or PET/CT fulfills the criteria of bone disease and, therefore, of symptomatic MM requiring treatment [4].

This review article provides an overview of the position of PET/CT in MM management with focus on the most widely used tracer ^18^F-FDG. In addition, the main data published on new PET tracers targeting different molecular pathways involved in MM pathogenesis are presented.

## 2. ^18^F-FDG PET/CT in MM

PET/CT is a whole-body imaging technique combining the functional information of PET with the morphological assessment provided by CT. ^18^F-FDG, the workhorse of PET imaging, is a biomarker of intracellular glucose metabolism. The tracer is actively transported into cells by the glucose transporter proteins (GLUT), which are expressed at a high degree in tumor cells due to their enhanced glucose demands. ^18^F-FDG, as a glucose analogue, is taken up by the neoplastic cells, undergoes phosphorylation and then gets trapped intracellularly, since ^18^F-FDG is not a substrate for further metabolic processing by either phosphohexose isomerase or glucose-6-phosphate dehydrogenase [6].

^18^F-FDG PET/CT has become nowadays a standard imaging technique in several tumor entities. Due to its ability in providing whole-body evaluations in a single session, the modality can assess the extent of oncological disease in a satisfying manner. In MM in particular, PET/CT can detect with a high sensitivity and specificity both medullary and extramedullary lesions [7]. Another important advantage of PET is the potential of quantification of tracer uptake by means of the index standardized uptake value (SUV), which reflects the amount of tracer activity in a particular region of interest. This quantification of tracer uptake aids in objective interpretation of PET/CT scans in addition to obtaining cross-sectional imaging and assessing ^18^F-FDG uptake visually, particularly in terms of patient follow-up. Furthermore—and most importantly—^18^F-FDG PET/CT can assess the metabolic burden and activity of MM in different stages of the disease due to its ability in differentiating between metabolically active and inactive lesions, with significant implications in treatment response assessment [5,7].

### 2.1. ^18^F-FDG PET/CT in the Diagnosis and Staging of MM

**Figure 1:**
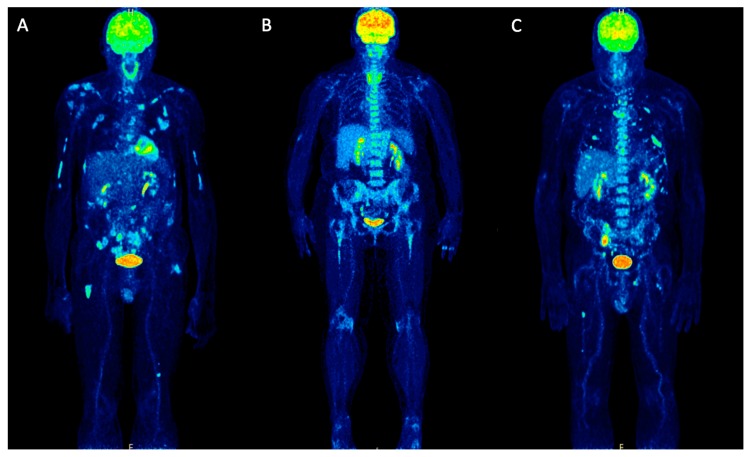
Maximum intensity projection (MIP) PET/CT images of newly diagnosed MM patients before treatment, representing examples of different pathologic patterns of ^18^F-FDG uptake. (**A**) demonstrates a patient with multiple focal lesions in the skeleton. (**B**) depicts a patient with intense diffuse tracer uptake in the bone marrow of the axial skeleton and the proximal humeri and femora without clearly delineated focal lesions. (**C**) shows a patient with a mixed pattern of ^18^F-FDG uptake with intense, diffuse uptake in the axial skeleton and multiple, focal bone marrow lesions.

^18^F-FDG PET/CT has been proven to be a very useful modality for the whole-body evaluation of the active burden of MM. Its reported sensitivity and specificity for assessment of medullary and extramedullary disease extent ranges from 80–100% [7,8,9,10,11,12]. The uptake pattern, SUV and different pharmacokinetic parameters of ^18^F-FDG correlate with the percentage of bone marrow plasma cells [13] (Figure 1).

PET/CT has been compared with other imaging modalities and has been shown to be superior to WBXR and comparable to MRI. In particular, a prospective study comparing ^18^F-FDG PET/CT with WBXR and pelvic-spinal MRI highlighted the superiority of PET/CT to WBXR in 46% of cases (sensitivity 92% vs. 61%). The sensitivity of PET/CT in the spine was inferior to MRI, underestimating the disease in a third of the patients; however, ^18^F-FDG PET/CT detected sites of active disease in areas outside the field of the MRI view [8]. Similarly, the results of a systematic review of 18 studies comparing the above-mentioned modalities showed a higher sensitivity of MRI at detecting diffuse disease of the spine, while ^18^F-FDG PET/CT was more sensitive than WBXR with regard to detection of bone lesions [10]. In another systematic review of 17 studies no significant differences were found between ^18^F-FDG PET/CT (sensitivity 91%, specificity 69%) and MRI (sensitivity 88%, specificity 68%) regarding detection rate of bone disease [11]. Recently, the prospective French IMAJEM study revealed no difference in the detection of bone lesions at diagnosis when comparing PET/CT and MRI with the former being positive in 95% and the latter in 91% of the patients [12].

Interestingly, there is a lack of studies regarding the comparison of ^18^F-FDG PET/CT with whole-body CT. According to the recently published consensus statement by the IMWG, although whole-body low-dose CT is the preferred method for the detection of lytic bone lesions in MM, ^18^F-FDG PET/CT should be considered as a valuable option, because of its ability to identify lytic lesions and extramedullary masses. Moreover, in cases of WBXR-negativity and whole-body MRI-unavailability, ^18^F-FDG PET/CT is recommended for the differentiation between active and smoldering MM [7].

Further, the newly emerging, hybrid PET/MRI technique seems highly attractive in the diagnostic approach of MM since it combines two modalities with a high potential in myeloma evaluation in a single exam. The results of the only prospective study comparing PET/CT with PET/MRI demonstrated good image quality provided by PET/MRI and high correlation between the modalities regarding the number of detected active lesions and SUV values [14]. However, further studies are warranted to evaluate the potential role of this novel technique in the diagnostics and management of MM.

### 2.2. Prognostic Value of ^18^F-FDG PET/CT in MM

^18^F-FDG PET/CT is a reliable outcome predictor and is regarded as the elective technique for treatment response evaluation of MM due to its ability to distinguish active from inactive sites of disease [9,12,15]. In newly diagnosed, symptomatic MM patients, three independent PET factors have been recognized to affect both progression-free survival (PFS) and overall survival (OS) in different prospective studies. These parameters are the number of focal, ^18^F-FDG-avid lesions, the SUV_max_ of the lesions, and the presence of EMD. Bartel et al. were the first to show in a group of 239 MM patients treated upfront with novel agents and double autologous stem-cell transplantation (ASCT) that the presence of more than three ^18^F-FDG-avid focal lesions was related to fundamental features of myeloma biology and genomics and was the leading independent parameter associated with inferior PFS and OS [9]. A few years later, in a study by Zamagni et al., including 192 MM patients treated with thalidomide-dexamethasone induction therapy and double ASCT, it was shown that the presence at baseline of at least three focal lesions, a SUV_max_ > 4.2 of the hottest lesion, and the presence of EMD adversely affected 4-year estimates of PFS, while SUV_max_ > 4.2 and EMD were also correlated with shorter OS [15]. Further, the IMAJEM study highlighted the role of EMD as an independent, adverse prognostic factor for both PFS and OS in 134 patients receiving a combination of lenalidomide, bortezomib, and dexamethasone with or without ASCT, followed by lenalidomide maintenance [12]. The prognostic significance of the three established PET risk factors was recently confirmed in a prospective study of 48 MM patients treated with induction treatment and ASCT. In that study it was also shown that not only quantitative PET parameters from focal lesions, but also those from reference bone marrow samples, are associated with adverse PFS in the disease [16].

Apart from its predictive role in symptomatic MM, ^18^F-FDG PET/CT has shown prognostic value in asymptomatic SMM patients. Although existing data are relatively limited, the first published results reflect the potential role of the modality in predicting the risk of progression from SMM to symptomatic disease. Siontis et al. studied a group of 122 SMM patients and found that the 2-year risk of progression to active MM was 75% in patients with a positive PET/CT (with or without lytic lesions), compared to 30% in patients with a negative PET/CT. The median time to progression (TTP) was 21 months for the PET/CT positive group, while the respective TTP for the PET/CT negative group was 60 months [17]. In another prospective, multicentric study of 120 SMM patients and a median follow-up of 2.2 years, patients with a positive PET study without underlying osteolysis had a higher risk of progression to active MM and a shorter TTP than patients who were PET-negative. In particular, 58% of the patients with a positive PET scan progressed to active myeloma in 2 years with a median TTP of 1.1 years, compared to those with a negative PET scan demonstrating a progression rate of 33% and a median TTP of 4.5 years [18].

### 2.3. The Value of ^18^F-FDG PET/CT in Therapy Assessment

**Figure 2:**
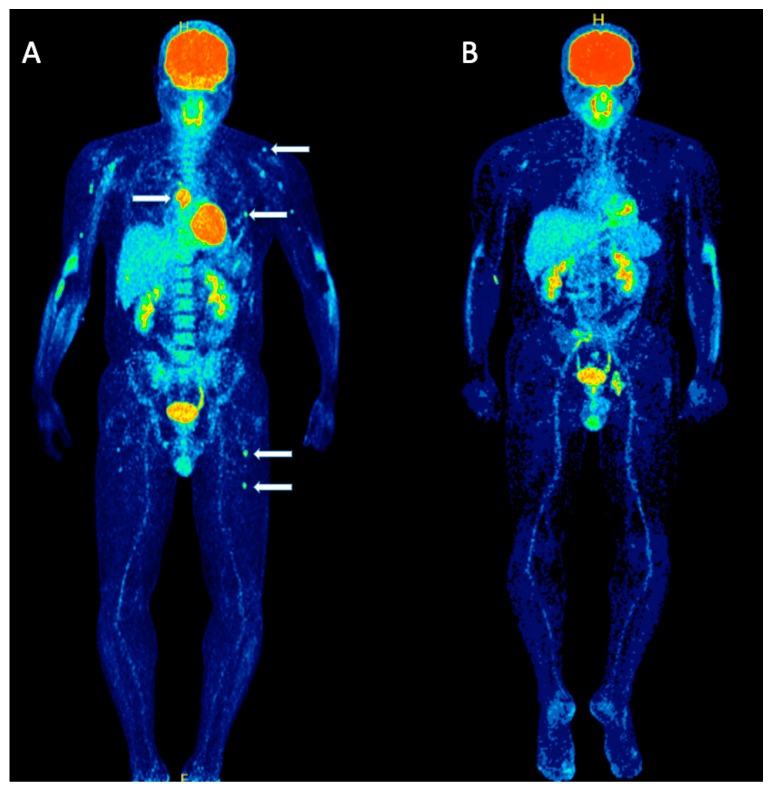
A 39-years old symptomatic MM patient scheduled for HDT and ASCT, undergoing ^18^F-FDG PET/CT before and after therapy. Maximum intensity projection (MIP) ^18^F-FDG PET/CT before therapy (**A**) revealed a mixed pattern of ^18^F-FDG uptake with intense, diffuse uptake in the axial skeleton and multiple, focal bone marrow lesions for example in the sternum, ribs, humerus, scapula and femur (arrows). Follow-up ^18^F-FDG PET/CT MIP after high-dose chemotherapy and ASCT (**B**) demonstrated a complete remission of both diffuse bone marrow uptake as well as focal MM lesions.

Due to its ability in distinguishing between active and inactive lesions, ^18^F-FDG PET/CT is the best imaging tool for therapy response assessment and is considered the gold standard for treatment monitoring in MM [7] (Figure 2). Several studies have highlighted the role of the modality in the evaluation of the metabolic response to therapy in different stages of the treatment protocol, for example during induction treatment as well as after ASCT [9,12,15,19,20,21,22,23].

In a study published by the Little Rock group in 2009 involving 239 previously untreated MM patients, it was shown that complete ^18^F-FDG suppression in focal lesions and EMD after induction treatment and before ASCT conferred superior OS and PFS, and was identified as an independent favorable prognostic variable [9]. A few years later, the same group published a study on a larger cohort involving 302 MM patients studied with PET/CT on day 7 of induction treatment. The authors showed that the persistence of more than three ^18^F-FDG-avid lesions imparted inferior OS and PFS, suggesting a therapy change in patients with persistent findings on PET/CT early after induction therapy [19]. Most recently, this team published the findings of a trial in 596 patients examined with PET/CT at different time points (day 7 of induction, end of induction, post transplantation, and at maintenance treatment). They demonstrated that patients achieving complete suppression of ^18^F-FDG activity in focal lesions following treatment at each studied time point had nonsignificant differences in their PFS and OS values than the patients with no lesions at baseline. Importantly, at each time point, patients with no detectable lesions had a significantly superior outcome compared to patients with at least one detectable lesion at that time point, irrespective of whether they had lesions at baseline [22].

The Bologna group has also highlighted the importance of ^18^F-FDG PET/CT in assessment of response to therapy in MM in different time points. In particular, they have shown that the persistence of severe ^18^F-FDG uptake—as reflected by the number of focal lesions, SUVmax and presence of EMD—after thalidomide/dexamethasone induction therapy is an early predictor of the worst long-term clinical outcomes. Moreover, a complete response (CR) on PET/CT after ASCT conferred superior PFS and OS in comparison with persistence of ^18^F-FDG uptake, while the prognostic value of PET/CT was retained also at the time of relapse, with patients positive on PET/CT having a significantly shorter survival compared to those with a negative PET/CT scan [15]. A few years later, they showed in a group of 282 patients that attainment of PET/CT negativity by 3 months after the last cycle of first-line treatment (chemotherapy, novel agents with or without ASCT) significantly influenced both PFS and OS [21]. PET/CT has also been shown effective in response evaluation of patients undergoing allogeneic stem cell transplantation with persistence of EMD being an independent predictor of poor outcome and, on the other hand, achievement of CR on PET/CT after transplantation being associated with a significantly longer OS [23].

The French group (IMAJEM trial) recently evaluated the role of PET/CT after induction treatment (lenalidomide, bortezomib, and dexamethasone) as well as before lenalidomide maintenance in a group of 134 MM patients. The authors showed that normalization of PET/CT after three cycles of induction therapy was associated with improved PFS, and that normalization before maintenance resulted in longer PFS and OS, in comparison to patients without normalization of their PET findings [12]. They could, moreover, show that change in SUV after three cycles of induction therapy was an independent prognostic factor for PFS, rendering SUV a potentially powerful tool for the prediction of long-term outcome in MM [24].

Other groups have also studied ^18^F-FDG PET/CT in the treatment response evaluation of MM, using different therapeutic agents and protocols. Most of them have confirmed the benefit of applying the modality in the workup of MM patients [25,26,27].

### 2.4. The Value of ^18^F-FDG PET/CT in Minimal Residual Disease (MRD) Diagnostics

A field that is constantly drawing more attention in MM therapy assessment is that of standardization and optimization of minimal residual disease (MRD) detection, which is becoming standard diagnostic care. This is driven by the need to improve the definition of disease remission due to the unprecedented rates of CR brought in recent years by the incorporation of novel agents in the treatment of MM patients. It is clear that in MM there is a direct correlation between the depth of response and prolonged survival rates [28]. At present, MRD is detected within the bone marrow, either by multicolor flow cytometry (MFC) or by next generation sequencing technologies [29].

Data on the potential role of ^18^F-FDG PET/CT in evaluation of the depth of response—beyond the level of conventionally defined CR- are limited but growing. Zamagni et al. retrospectively analyzed 282 MM patients who were evaluated at baseline and during posttreatment follow-up with serial PET/CT scans. They found that the modality could provide a more accurate definition of CR, allowing to stratify patients in conventional CR after up-front therapy into different prognostic subgroups, according to the persistence or absence of ^18^F-FDG metabolic activity. In particular, the achievement of PET-negativity after treatment was an independent predictor of prolonged PFS and OS for patients with conventionally defined CR [21]. Furthermore, the complementary role of PET/CT and MRD diagnostics with MFC in predicting patient outcome has been supported by some studies. A subanalysis of the IMAJEM trial in 86 patients before maintenance evaluated for both PET/CT and MRD, assessed by MFC, revealed a higher PFS for the group of patients with both a normalized PET/CT and a negative MRD versus patients with either PET positivity and/or MRD positivity before maintenance [12]. In line with these results, the Little Rock group showed in 83 MM patients in CR with available MRD and functional imaging data (in this case PET/CT and/or diffusion weighted MRI) that double-positive and double-negative features defined groups with dismal and excellent PFS, respectively [30]. Most recently, a retrospective study analyzed the prediction of outcome with the combination of ^18^F-FDG PET/CT and MRD, assessed by MFC, in 103 patients with newly diagnosed MM. Apart from confirming the benefit—in terms of PFS—linked to the achievement of negativity by MFC and ^18^F-FDG PET/CT individually, the authors showed that the combination of negativity by both techniques conferred significantly higher PFS than each technique alone, also supporting the potential complementarity between PET/CT and MFC in MRD detection [31].

### 2.5. Limitations of ^18^F-FDG PET/CT

Limitations of ^18^F-FDG PET/CT include its limited availability in comparison to conventional radiological modalities as well as its higher cost. Moreover, the poor sensitivity for the detection of diffuse bone marrow infiltration or skull lesions, due to masking of their activity by the underlying physiological tracer uptake in the brain, is an important drawback. In a report of 227 MM patients the incidence of PET false-negativity was 11% in these patients, a finding attributed to the significantly lower expression of the gene coding for hexokinase-2, which catalyzes the first step of glycolysis [32]. However, this explanation warrants further validation [33]. Further, ^18^F-FDG, as a glucose analog, is generally restricted in oncological imaging by both false positive (inflammation, post-surgical areas, recent use of chemotherapy, fractures, etc.) and false negative results (hyperglycemia, recent administration of high-dose steroids, etc.). Finally, issues are raised due to the lack of established criteria for image interpretation of ^18^F-FDG PET/CT scans in MM, resulting in poor interobserver reproducibility in interpreting results. In an attempt to standardize the interpretation of ^18^F-FDG PET/CT, the Bologna group has recently proposed the Italian Myeloma criteria for PET Use (IMPeTUs) based on the standard Deauville five-point system [34]. These descriptive criteria take into account the number and site of focal lesions, the presence of EMD, as well as the diffuse bone marrow involvement. The first results from the application of IMPeTUs seem to improve the interobserver reproducibility in scan interpretation; however, this needs to be confirmed in further studies.

## 3. Non ^18^F-FDG PET Tracers in MM

Due to the limitations of ^18^F-FDG as an imaging biomarker of MM, several other PET tracers have been proposed and tested in patients with the malignancy. Although some of them have given promising results regarding detection of MM lesions, most studies were performed in rather small patient cohorts and, thus, require validation in further prospective clinical trials. The most important of them will be addressed in the following paragraphs.

### 3.1. ^18^F-Choline and ^11^C-Choline

Choline is a component of phosphatidylcholine and, as such, functions as a substrate for cell membrane biosynthesis. The uptake of radiolabeled choline is increased in proliferating cells because it is involved in membrane metabolism and growth. Choline PET imaging has been traditionally used in the diagnostics of prostate cancer.

The first report of ^11^C-choline uptake in myeloma lesions was an incidental finding of a solitary plasmacytoma in a patient being re-staged for prostate cancer [35]. Based on this finding, a comparison study of ^11^C-choline vs. ^18^F-FDG PET/CT in assessing bone involvement was performed by the Bologna group in a heterogeneous group of 10 MM patients (4 patients at completion of initial therapy, 2 during follow-up and 4 at disease relapse). In 2/10 patients with suspicion of disease relapse, both the ^11^C-Choline and ^18^F-FDG PET/CT scans were positive and identified the same number and sites of bone lesions. In 4/10 patients, both techniques were positive, but ^11^C-choline identified a nonsignificant higher number of lesions than ^18^F-FDG. Finally, 4 patients were negative with both tracers, a finding consistent with clinical, laboratory and radiological data indicating a CR at the time of imaging [36]. Almost ten years later, another pilot study on choline PET was published on a larger MM patient cohort. Twenty-one patients with suspected progressive or relapsing MM were studied with ^18^F-choline and ^18^F-FDG PET/CT. No myeloma lesions were detected in two cases, while uncountable foci were observed in four patients. In the rest, 15 patients with countable bone foci, ^18^F-choline PET/CT depicted a significantly higher number of lesions than ^18^F-FDG PET/CT [37]. Further, the performance of ^18^F-choline and ^18^F-FDG PET/CT in the detection of skeletal involvement was compared in a case series of five MM patients in a pairwise fashion. Skeletal lesions were detected in all five ^18^F-choline PET/CT scans compared to four out of five ^18^F-FDG PET/CT scans. Altogether ^18^F-choline PET/CT detected a total of 134 bone lesions compared to 64 lesions detected by ^18^F-FDG PET/CT. Interestingly, the vast majority of the missed lesions in ^18^F-FDG PET/CT were in the axial skeleton including the skull vault [38].

To summarize, choline PET seems to have a better detection rate of focal lesions than ^18^F-FDG PET. However, no comparison studies between the two PET tracers in previously untreated MM patients have been performed. A limitation of choline PET is its unfavorable physiological distribution involving increased uptake in the bone marrow and the liver parenchyma potentially masking lesions in these organs; although hepatic lesions are rare in MM and can be reliably detected with MRI, the increased activity in the bone marrow compartment may pose significant diagnostic challenges, in particular in patients showing a diffuse bone marrow infiltration pattern. Moreover, the use of ^11^C-choline is limited in centres with an on-site cyclotron and radiopharmacy facilities, because of the very short half-life of the radioisotope (20 min).

### 3.2. ^11^C-Acetate

^11^C-acetate is rapidly picked-up by cells and metabolized into acetyl-CoA by the key enzyme acetyl-CoA synthase, which is overexpressed in certain cancer cells [39]. The use of ^11^C-acetate in MM can be justified by the elevated lipid synthesis in proliferating abnormal plasma cells as reported by studies with myeloma cell lines [40].

Similarly to radiolabeled choline, the first report of ^11^C-acetate uptake in myeloma lesions was an incidental finding [41]. In total, two comparative studies of ^11^C-acetate with ^18^F-FDG have been published thus far. Ho et al. evaluated a heterogeneous group of 35 untreated patients (26 with symptomatic MM, 5 with SMM, and 4 with MGUS), 9 of which undergoing also dual tracer follow-up PET/CT. The authors reported a significantly higher overall sensitivity for symptomatic MM with ^11^C-acetate than with ^18^F-FDG (84.6% vs. 57.7%), while the specificity for ^11^C-acetate and ^18^F-FDG PET/CT was 100% and, 93.1% respectively. Furthermore, all indolent plasma cell neoplasms (SMM and MUGS) were negative by ^11^C-acetate PET, whereas 2 cases of MGUS were false-positive by ^18^F-FDG [42]. A similar study was published a few months later by Lin et al. in 15 untreated MM patients examined with both tracers at diagnosis, 13 of which being evaluated with a repeated dual-tracer examination after completion of induction treatment. They found a higher detection rate for both diffuse and focal myeloma lesions at initial staging using ^11^C-acetate than ^18^F-FDG. Moreover, after treatment the diffuse bone marrow ^11^C-acetate uptake showed a statistically significant difference in SUV_max_ reductions between patients with at least a very good partial response and those with at most a partial response. Such a difference between patients in these two response groups was not observed with ^18^F-FDG PET/CT [43].

In summary, these preliminary findings imply a potential role for ^11^C-acetate PET/CT for the evaluation of patients with MM. Nevertheless, practical and logistical considerations are raised due to the fact that the synthesis of the tracer requires technical expertise and an on-site cyclotron.

### 3.3. ^11^C-Methionine

^11^C-Methionine is an aminoacidic PET tracer mainly employed in the diagnosis of central nervous system tumors. The uptake of the tracer primarily reflects its transmembrane transport by the sodium-independent L-transporter into cells. This transport is driven by concentration gradient and is thus influenced by the intracellular metabolism of the amino acid, which in turn reflects proliferation activity [44]. The concept of applying ^11^C-methionine in MM is mainly based on the knowledge that radiolabeled amino acids show a rapid uptake and metabolic incorporation into newly synthesized immunoglobulins [45]. Moreover, the uptake of 35S-methionine into myeloma cells is higher as compared with other hematopoietic cells [46]. Despite the limited literature on the topic, ^11^C-methionine PET/CT concordantly appears to perform better than ^18^F-FDG in detection of myeloma lesions.

Dankerl et al. were the first to apply this PET tracer for imaging of MM in a group of 19 patients with active disease. The authors detected disseminated multifocal ^11^C-methionine–positive bone marrow lesions in all patients, except two, a finding suggesting widespread dissemination of MM in the hematopoietic bone marrow. The two patients without extensive disease on ^11^C-methionine PET showed exclusive EMD and monofocal medullary MM, respectively [46]. The first comparative study was published in 2013 by Nakamoto et al. in 20 patients with MM (n = 15) and plasmacytoma (n = 5) who underwent ^18^F-FDG PET/CT and ^11^C-methionine PET/CT scans. On a patient basis, two patients were accurately diagnosed only by ^11^C-methionine PET/CT, while in the remaining 18 patients consistent results were obtained. However, the potential upgrade of staging or restaging was necessary in 6 of 11 positive patients because more abnormal lesions were demonstrated by ^11^C-methionine PET/CT. The patient-based sensitivity, specificity and accuracy of ^11^C-methionine PET/CT for restaging were 89%, 100% and 93%, respectively, while those of ^18^F-FDG PET/CT were 78%, 100% and 86%, respectively [47]. Two years later, Okasaki et al. studied 64 patients with MM or MGUS (21 previously untreated, 43 restaged after treatment) undergoing PET/CT with the tracers ^11^C-4′-thiothymidine (^11^C-4DST), ^11^C-methionine, and ^18^F-FDG. The main findings of the study were the following: firstly, the number of equivocal lesions observed using ^18^F-FDG was larger compared to using ^11^C- methionine or ^11^C-4DST both before and after therapy. Secondly, ^11^C- methionine and ^11^C-4DST were superior to ^18^F-FDG in clearly detecting skull lesions because of their low physiological accumulation in the brain [48].

The Würzburg group has also highlighted the superiority of ^11^C-methionine over ^18^F-FDG for staging and re-staging of both intra- and extramedullary MM lesions [49,50]. These results were further confirmed in both patient- and lesion-based analyses in the largest so far, dual-center study of 78 patients (4 solitary plasmacytoma, 5 SMM, 69 symptomatic MM) published in 2017 [51]. Moreover, the same group has recently performed the first head-to-head comparison of ^11^C-methionine and ^11^C-choline for metabolic imaging of MM in 19 patients with a history of MM (n = 18) or solitary bone plasmacytoma (n = 1). ^11^C-methionine provided advantages over ^11^C-choline in terms of higher sensitivity by detecting a higher number of intramedullary lesions in approximately 40% of patients, as well as by achieving higher lesion-to-background contrast [52].

Drawbacks of ^11^C-Methionine PET are considered to be its increased physiological biodistribution in the liver parenchyma and the bone marrow, potentially reducing the detection rate of MM lesions. Moreover, the ^11^C labeling of the tracer prevents a relatively massive production and distribution of ^11^C-Methionine [53].

### 3.4. ^18^F-Fluorothymidine (^18^F-FLT)

^18^F-Fluorothymidine (^18^F-FLT) is the most studied cellular proliferation PET agent [54]. ^18^F-FLT is taken up by cells and phosphorylated by thymidine kinase 1, which is upregulated by about tenfold during the S-phase of the cell cycle, producing ^18^F-FLT monophosphate (^18^F-FLT-MP), which can then be sequentially phosphorylated to form ^18^F-FLT diphosphate (^18^F-FLT-DP) and ^18^F-FLT triphosphate (^18^F-FLT-TP). These phosphorylated products are metabolically trapped intracellularly without being incorporated into DNA. The tracer retention within cells reflects, in part, thymidine kinase activity and is often positively correlated with cellular proliferation [55].

The knowledge regarding application and performance of ^18^F-FLT PET in MM is limited. Agool et al. studied a group of 18 patients with different hematologic disorders, among which were two patients with MM. The authors found that the affected osteolytic areas in these two MM patients demonstrated a low ^18^F-FLT uptake [56]. In a pilot study on combined ^18^F-FDG and ^18^F-FLT PET/CT imaging in 8 myeloma patients (4 patients with symptomatic MM, 4 patients with SMM) the number of myeloma-indicative lesions was significantly higher for ^18^F-FDG PET/CT than for ^18^F-FLT PET/CT. A common finding of the study was a mismatch of focally increased ^18^F-FDG uptake and reduced ^18^F-FLT uptake (lower than the surrounding bone marrow) in myeloma lesions. Moreover, ^18^F-FLT PET/CT was characterized by high background activity in the bone marrow compartment, complicating the evaluation of bone marrow lesions [57].

In conclusion, despite the limited number of patients studied so far, the preliminary results indicate that ^18^F-FLT does not seem suitable as a single PET tracer in MM diagnostics.

### 3.5. ^68^Ga-Pentixafor

Chemokine receptor 4 (CXCR4) is a pleiotropic, G-protein coupled chemokine receptor expressed on hematopoeitic stem and progenitor cells in the bone marrow niche. CXCR4 can mediate the migration as well as the homing process of these cells in the bone marrow in response to its ligand, stromal cell-derived factor 1 (SDF-1) [58]. In MM, CXCR4 is involved in myeloma cell homing, bone marrow retention, angiogenesis and metastasis, while collective evidence from several studies support the pivotal role of CXCR4 in different stages of MM, disease progression, development of therapeutic resistance and MRD, as well as poor prognosis [59,60,61,62,63,64,65,66].

^68^Ga-pentixafor is a radiolabeled peptide that shows high affinity for CXCR4. The major advantage of the tracer is its potential use in a thera(g)nostic approach in combination with the ^177^Lu- or ^90^Y-labeled agent pentixather in progressive MM patients with CXCR4-positive tumor cells, as confirmed by a ^68^Ga-pentixafor PET scan. Preliminary results of the CXCR4-directed endoradiotherapy with pentixather in three heavily pretreated MM patients were relatively encouraging with low levels of toxicity, good tolerance of the treatment and high initial response rates [67].

Two studies have investigated the diagnostic performance of ^68^Ga-pentixafor in comparison to ^18^F-FDG in patients with advanced MM. The initial results in 14 MM patients showed a slight superiority of the novel tracer over ^18^F-FDG in the relapsed disease setting, with 10/14 patients showing MM manifestations on ^68^Ga-pentixafor PET, while 9/14 were positive on ^18^F-FDG PET [68]. The larger second study included 35 patients undergoing ^68^Ga-pentixafor PET/CT for evaluation of eligibility for endoradiotherapy. In 19 patients, ^18^F-FDG PET/CT was also available for correlation. ^68^Ga-pentixafor PET detected CXCR4-positive disease in 23/35 subjects (66%). Importantly, in the 19 patients in whom a comparison to ^18^F-FDG PET was available, 8/19 (42%) patients had an equal number of lesions with both tracers, in 4/19 (21%) subjects ^68^Ga-pentixafor PET detected more lesions, while ^18^F-FDG PET proved superior in 7/19 (37%) of them [69].

Most recently, the first comparative study of ^18^F-FDG and ^68^Ga-pentixafor PET/CT in 30 patients with newly diagnosed MM was published. ^68^Ga-Pentixafor PET/CT had a significantly higher positive rate than ^18^F-FDG PET/CT in detection of myeloma lesions (93.3% vs. 53.3%). In quantitative analysis, bone marrow uptake values in ^68^Ga-Pentixafor were positively correlated with end organ damage, staging, and laboratory biomarkers related to tumor burden including serum β2-microglobulin, serum free light chain, and 24-h urine light chain. In contrary, in ^18^F-FDG PET/CT, only the SUV mean of total bone marrow was positively correlated with serum free light chain and 24-h urine light chain [70]. These results indicate that ^68^Ga-pentixafor PET might be a promising biomarker in assessing the tumor burden of newly diagnosed MM patients.

### 3.6. ^18^F-Sodium Fluoride (^18^F-NaF)

^18^F-NaF is a highly sensitive biomarker of bone reconstruction, with potential indications in a wide range of bone disease [71,72,73,74]. The uptake of the tracer in bone occurs by chemisorption onto hydroxyapatite, followed by exchange with hydroxyl groups in the hydroxyapatite, resulting in formation of fluoroapatite. The tracer accumulates in nearly all sites of increased new bone formation, reflecting regional blood flow, osteoblastic activity and bone turnover [71,75,76].

An increasing interest has been raised in the last years on the potential application of ^18^F-NaF PET/CT in MM diagnostics and management. This interest was based, however, on a very small number of studied MM patients without comparison with a robust reference imaging method [77,78,79,80]. Despite this initial enthusiasm, subsequent publications demonstrated rather discouraging results. In particular, ^18^F-NaF PET/CT did not confer any superiority or complementarity to ^18^F-FDG PET/CT in detection of MM lesions, showing both lower sensitivity and specificity [81,82,83]. Moreover, ^18^F-NaF PET/CT does not seem to add significantly to ^18^F-FDG PET/CT in the treatment response evaluation of MM patients, as shown in a study of 34 patients undergoing high-dose chemotherapy and ASCT [84].

The low sensitivity of ^18^F-NaF PET/CT in detecting myeloma lesions is mainly attributed to the fact that the tracer indicates osteoblastic activity. However, since the hallmark of MM is the osteolytic lesion, the accumulation of ^18^F-NaF takes place only in the accompanying, sometimes minimal, reactive osteoblastic changes [85]. Further, being a very sensitive radiopharmaceutical for osteoblastic activity, ^18^F-NaF accumulates in practically in every site of newly mineralizing bone, irrelevant of its aetiology. This means that any cause of bone reconstruction, such as traumatic or degenerative bone lesions, will lead to tracer accumulation, significantly decreasing its specificity as a myeloma tracer [86].

### 3.7. ^18^F-FAZA

One of the reasons leading to an increased metabolic activity detected with ^18^F-FDG PET/CT is tumor hypoxia. Tumor hypoxia leads to enhanced production of several hypoxia inducible factors, resulting in increased microvessel density (MVD) around the malignant plasma cells [6]. MVD has been proven to be correlated with disease progression in MM [87]. Based on this approach, de Waal et al. applied the PET tracer 1-α-D: -(5-deoxy-5-[^18^F]-fluoroarabinofuranosyl)-2-nitroimidazole (^18^F-FAZA), which accumulates in tumor hypoxia. The authors studied 5 patients with relapsed MM with ^18^F-FDG PET and ^18^F-FAZA PET. Although all patients had a positive ^18^F-FDG PET scan, no lesions were demonstrated on ^18^F-FAZA PET, reflecting a limited performance of this tracer in the workup of MM patients [88].

### 3.8. ^89^Zr-Daratumumab

The membrane glycoprotein cluster of differentiation 38 (CD38) is expressed at a high density by almost all myeloma cells, and at relatively low levels on normal hematopoietic cells. CD38 is an established therapeutic target in MM. Daratumumab is an FDA-approved therapeutic monoclonal antibody that binds directly to CD38, offering a clinical benefit in MM patients [89,90,91]. Recently, daratumumab was radiolabeled with ^89^Zr through deferoxamine (DFO), producing the PET agent ^89^Zr-DFO-daratumumab. The results of a Phase I first-in-human ^89^Zr-DFO-daratumumab PET/CT imaging study in six MM patients demonstrated successful whole-body PET visualization of MM with focal tracer uptake in previously known as well as unknown sites of osseous myeloma, consistent with successful CD38-targeted immunoPET imaging of myeloma in human patients [92]. Although these results warrant validation in further prospective studies, they are highly promising for the usage of this PET antibody in diagnosis and staging of MM. Moreover, it could be applied in terms of a personalized, daratumumab-directed imaging in order to identify those MM patients who would benefit from daratumumab and thus predict the effectiveness of therapy in the context of a thera(g)nostic approach in MM.

## 4. Conclusions

PET/CT with ^18^F-FDG is increasingly gaining acceptance in the management of MM patients, and is considered a powerful diagnostic tool for the detection of medullary and extramedullary disease at initial diagnosis, a reliable predictor of survival, as well as the most robust modality for treatment response evaluation in the disease. On the other hand, ^18^F-FDG carries the limitations of a rather poor sensitivity for the detection of diffuse bone marrow infiltration, a relatively low specificity, and the lack of widely applied, established criteria for image interpretation. These drawbacks have led to the development of several alternative PET tracers for MM detection. Some of these radiotracers have provided promising results—such as ^18^F-choline and ^11^C-choline, ^11^C-acetate, ^11^C-methionine, ^68^Ga-pentixafor and ^89^Zr-Daratumumab—but most studies were performed in small patient cohorts and require validation in further prospective clinical trials.
